# Whole-Genome Sequence of *Streptococcus macedonicus* Strain 33MO, Isolated from Curd of Morlacco Cheese in the Veneto Region (Italy)

**DOI:** 10.1128/genomeA.00746-14

**Published:** 2014-08-07

**Authors:** Veronica Vendramin, Laura Treu, Barbara Bovo, Stefano Campanaro, Viviana Corich, Alessio Giacomini

**Affiliations:** a Department of Agronomy, Food, Natural Resources, Animals and Environment (DAFNAE), University of Padova, Padua, Italy; b Department of Environmental Engineering, Technical University of Denmark, Kongens Lyngby, Denmark; c Department of Biology and CRIBI Biotechnology Centre, University of Padova, Padua, Italy

## Abstract

A genetic characterization of *Streptococcus macedonicus* is important to better understand the characteristics of this lactic acid bacterium, frequently detected in fermented food bacteria communities. This report presents the draft genome sequence description of strain 33MO, the first publicly available genome sequence of an Italian *S. macedonicus* isolate.

## GENOME ANNOUNCEMENT

Thermophilic lactic acid bacteria (LABs) play essential roles in dairy fermentation, particularly in cheesemaking. Among these bacteria, *Streptococcus macedonicus*, ﬁrst isolated from Greek Kasseri cheese (1) and recently from Italian cheeses (2), might play a relevant role in the characterization of artisanal cheese flavors. To date only one complete genome sequence of *S. macedonicus* has been published (3). Morlacco is a traditional cheese of the mountain pastures around Mount Grappa, in northern Italy. Its intense aroma makes it an important element in the gastronomic tradition, and the characterization of LABs involved in its production is relevant for better understanding and improvement of the organoleptic properties of cheese. For this reason, *S. macedonicus* 33MO, a strain isolated from Morlacco cheese curd (2), was chosen for genome sequencing.

Genome sequencing was performed with an Illumina MiSeq sequencer at the Ramaciotti Centre, Sydney, Australia. Genomic libraries were prepared using a Nextera XT kit (Illumina, Inc., San Diego, CA, USA). A total of 2,102,017 paired-end reads (2 × 250) were generated and resulted in 230-fold coverage of the genome. Approximately 85% of these reads were assembled into 73 large scaffolds by use of a manually curated consensus of assemblies obtained using Velvet software v. 1.2.10 (4). The draft genome of *S. macedonicus* 33MO is 2,220,931 bases in length, with a mean GC content of 37.4%.

Genome annotation was performed by the RAST annotation server (5) and by NCBI’s Prokaryotic Genome Annotation Pipeline (6). A total of 2,355 coding sequences (CDS), distributed in 302 subsystems, and 52 structural RNAs were predicted. Moreover, 24 phage-associated sequences and only 4 clusters of regularly interspaced short palindromic repeats (CRISPRs) were identified.

Comparison with *S. macedonicus* ACA-DC198, the only other strain with a genome sequence available for this species, demonstrated that 33MO contains a number of bacteriocin-related sequences, confirming previous phenotypic characterization results (2). In addition, strain 33MO shows features related to amino acid metabolism pathways, in particular, genes related to arginine and ornithine degradation, which are known to be involved in secondary flavor compound biosynthesis in different food products, such as bread (7), cheese (8), and wine (9).

A genetic comparison with strains of the neighbor species *Streptococcus thermophilus*, isolated from a cheese environment, was performed to look for possible characteristics unique for this species. With respect to *S. thermophilus* (10–12), *S. macedonicus* possesses more carbohydrate pathway subsystems (from 237 to 251 CDS in the two *S. macedonicus* strains sequenced versus from 157 to 171 CDS in *S. thermophilus*), and presents major differences in di- and oligosaccharides utilization. *S. macedonicus* exhibits interesting fructooligosaccharide (FOS) and raffinose utilization proprieties, which are not present in the strains of the other species. Finally, *S. macedonicus* shows beta-glucoside metabolism, which may be useful in vegetable fermentation.

### Nucleotide sequence accession numbers.

This whole-genome shotgun project has been deposited at DDBJ/EMBL/GenBank under the accession JNCV00000000. The version described in this paper is version JNCV01000000.
